# Do patients want clinicians to ask about social needs and include this information in their medical record?

**DOI:** 10.1186/s12913-022-08652-5

**Published:** 2022-10-22

**Authors:** Steven M Albert, Polly McCracken, Thuy Bui, Janel Hanmer, Gary S Fischer, Jaishree Hariharan, Alton Everette James

**Affiliations:** 1 Department of Behavioral and Community Health Sciences, School of Public Health, University of Pittsburgh, 6116, 130 DeSoto St, Pittsburgh, USA; 2 Division of General Internal Medicine, Department of Medicine, University of Pittsburgh, Pittsburgh, USA; 3 Pitt Health Policy Institute and Department of Health Policy and Management, University of Pittsburgh, Pittsburgh, USA

**Keywords:** Social needs, EHR, Screening, Social determinants of health, Primary care

## Abstract

**Background:**

Social needs screening in primary care may be valuable for addressing non-medical health-related factors, such as housing insecurity, that interfere with optimal medical care. Yet it is unclear if patients welcome such screening and how comfortable they are having this information included in electronic health records (EHR).

**Objective:**

To assess patient attitudes toward inclusion of social needs information in the EHR and key correlates, such as sociodemographic status, self-rated health, and trust in health care.

**Design, participants, and main measures:**

In a cross-sectional survey of patients attending a primary care clinic for annual or employment exams, 218/560 (38%) consented and completed a web survey or personal interview between 8/20/20-8/23/21. Patients provided social needs information using the Accountable Care Communities Screening Tool. For the primary outcome, patients were asked, “Would you be comfortable having these kinds of needs included in your health record (also known as your medical record or chart)?”

**Analyses:**

Regression models were estimated to assess correlates of patient comfort with including social needs information in medical records.

**Key results:**

The median age was 45, 68.8% were female, and 78% were white. Median income was $75,000 and 84% reported education beyond high school. 85% of patients reported they were very or somewhat comfortable with questions about social needs, including patients reporting social needs. Social need ranged from 5.5% (utilities) to 26.6% (housing), and nonwhite and gender-nonconforming patients reported greater need. 20% reported “some” or “complete” discomfort with social needs information included in the EHR. Adjusting for age, gender, race, education, trust, and self-rated health, each additional reported social need significantly increased discomfort with the EHR for documenting social needs.

**Conclusions:**

People with greater social needs were more wary of having this information placed in the EHR. This is a concerning finding, since one rationale for collecting social need data is to use this information (presumably in the EHR) for addressing needs.

Recognizing the significance of the social determinants of health (SDOH), the Centers for Medicare and Medicaid Services has requested comment on two quality measures related to social risk or need. These include the rate of screening for social needs (% beneficiaries age 18 years and older screened for food insecurity, housing instability, transportation problems, utility help needs, and interpersonal safety) and the screen-positive rate [1]. If approved, the two measures may be included in the Merit-based Incentive Payment System and Hospital Inpatient Quality Reporting Program, which will encourage standardization in reporting and provide important incentives for screening. CMS has adopted the Accountable Health Communities (AHC) Health-Related Social Needs (HRSN) Screening Tool for this purpose [2]. These measures imply documentation of social needs in the electronic health record (EHR). While research suggests high acceptability of social needs screening, the acceptability of EHR documentation of these needs is less clear.

The prevalence of social needs in adult primary care is high. A large Kaiser Permanente Northwest study conducted in 2017-19 reported a prevalence of 24% for at least one social need [3]. More than half of patients reporting such needs also sought help to address the need, consistent with literature showing that 40–60% of people who report an unmet need agree to participate in patient navigation or other social needs programs [4]. Among members of the Kaiser Permanante health system who received federally subsized health insurance, 48% reported at least one “social risk factor” [5]. Across 19 ambulatory sites within a health system in the Bronx, NY, 20% of patients reported one or more social needs or risks [6]. A review found that 5-43% of participants screened in clinical settings had needs related to housing stability, 6-41% food-related needs, and 47-89% general needs related to financial insecurity [4].

Patients are willing to answer questions about social needs. About three-quarters of patients are willing to answer questions about income [7], for example, an important indicator of need. One study reported 81-95% completion of social needs screening questions, with 85% agreeing that a health system should ask patients about these needs [8]. Patients reporting a greater number of social needs were more likely to agree that social needs affect health and endorse clinician efforts to elicit needs and address them.

Yet support for collecting such information does not rule out discomfort with EHR documentation of social needs. One study recruited patients from six primary care clinics and four emergency departments in nine states and found high rates of screening acceptability (79%) but less support for including this information in electronic health records (65%) [9]. As the authors write, “more patients were comfortable with social screening itself than with its documentation in the EHR.” The authors conclude that “lack of patient acceptability should not be a major barrier to implementation of social risk screening” but also note that 19% reported being “very” or “somewhat uncomfortable” with EHR documentation of needs. Acceptability of EHR documentation was as low as 54% among subgroups of patients.

These findings suggest the need for a closer look at patient attitudes toward social need information and its documentation in the patient health record. For this reason, we investigated patient social needs and attitudes toward EHR documentation of these needs in an academic primary care clinic.

## Methods

### Sample

Between 8/20/20-8/23/21, we approached all scheduled patients seen in a single hospital-based primary care clinic for annual or employment examinations to ascertain interest in the study. This urban clinic is located in a large teaching hospital and is affiliated with the University of Pittsburgh. Faculty physicians are board-certified in internal medicine and supervise residents; two health educators, a dietitian, a social worker, and a pharmacist are available to support the medical team. Patients can view test results, schedule appointments, request prescription refills, and communicate with physicians and staff using a secure patient portal. All physicians receive 1 hour of mandatory training in communicating about culturally sensitive issues as well as additional training in addressing racism and implicit bias and diversity and inclusion.

Patients were approached by a medical assistant or physician who described the study using a prepared script. Potential participants were asked during a telemedicine or in-person appointment if they would be interested in participating in a study about “what it takes to be healthy and the role of housing, food security, medical transportation, utilities (heat, light, water), and personal safety.” Patients expressing interest provided contact information. The research team then followed up by telephone and email contact and obtained consent. Patients were not compensated for participation in the study. The study was approved by the University of Pittsburgh Human Research Protections Office.

The clinic is based at a university hospital and serves a diverse patient population, including unversity faculty and staff, hospital employees, and community members. It includes a residency program and sees patients with a variety of insurance coverage, including Medicaid.

### Design

Participants were invited to complete the AHC Health-Related Social Needs Screening (HRSN) a single time within 1-2 weeks of their clinic visits. We used the expanded questionnaire developed by De Marchis, et al. [9] Patients were paired with research assistants who followed up with potential participants to encourage completion of social needs screening either through an Internet survey sent via email or by telephone interview. Patient responses were de-identified and not linked to the EHR.

### Measures

To measure social needs, patients completed the Accountable Health Communities (AHC) Health-Related Social Needs Screening Tool [10]. CMS developed the 10-item screening tool to identify patient needs that can be addressed through community services. The measure assesses five domains (housing instability, food insecurity, transportation difficulties, utility assistance needs, and interpersonal safety).

The AHC HRSN can be scored in a variety of ways to indicate social needs. We used the following thresholds: *Unstable housing*: 1+ problem (pests, mold, lead, heat, oven, no smoke detector, water leak) or unstable housing (anxious about losing home or homeless). *Food insecurity*: Sometimes/often run out of food or food does not last until end of month. *Transportation need*: Lack of reliable transportation. *Intermittent utilities*: utilities shut off in past year. *Threat to safety*: Worried about safety, threatened with harm, or sometimes/often screamed at or cursed. We computed a sum for the number of social needs reported (range, 0-5).

We included a number of additional measures used by De Marchis et al. [9]. These elicit additional information on the five domains of social need as well as patient experience answering these questions: have they been asked the questions in the prior 12 months, have they received assistance with any of the needs, do they think it is appropriate to be asked such questions, and “would you be comfortable having these kinds of needs included in your health records (also known as your medical record or chart).” Other questions elicited a rating of health, trust in your health care provider (1-10 scale), and “where you see yourself in relation to others in the United States” (1, worst off-10, very top). Patients were also asked if they experienced disrespect from health care providers using a 6-point index. Finally, patients reported sociodemographic information including income categories (13-point scale ranging from <$5,000 to >=$150,000). The questionnaire is available online: https://www.ajpmonline.org/cms/10.1016/j.amepre.2019.07.010/attachment/f00500c1-4267-4be7-b089-f01328d38471/mmc1.pdf.

### Analyses

Analyses were mainly descriptive and sought to characterize the level and type of social needs reported by the sample along with additional indicators including whether patients were asked about each need in clinic visits, how comfortable they were with this line of questioning, whether they wanted assistance with the need, and whether they received assistance.

We compared means by t-test and computed Pearson and Spearman correlations. To assess the relationship between social needs and comfort with recording such information in the EHR, we estimated linear and ordinal regression models that adjusted for these factors to identify how much comfort with EHR documentation changed with each additional reported social need. Analyses were conducted using STATA/SE 15.1.

## Results

**Fig. 1:**
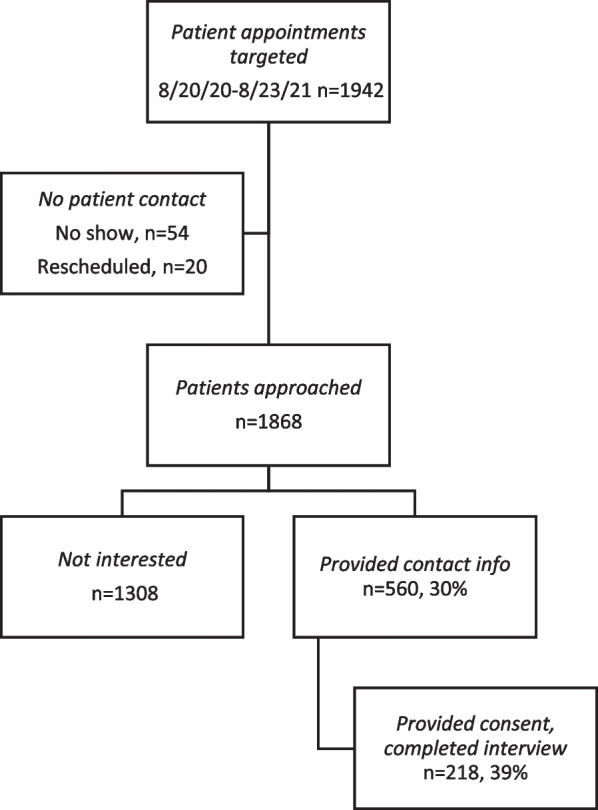
Patient recruitment

Of 1868 patients who were seen at the clinic for annual or employment exams over 12 months, 560 (30%) provided contact information after they were approached by a physician or medical assistant during the appointment (Fig. 1). Of these patients, 218 (39%) provided informed consent and completed the AHC HRSN by Internet survey (66%) or telephone interview (34%). Patients were seen by a total of 29 attending physicians and 20 residents. Despite the potentially sensitive nature of the interview and heavy use of a self-administered Internet questionnaire, the proportion with missing data was low. In the multiple regression analysis reported below, complete data were available for 202 (92.7%) respondents. We did not include income in the regression model because 15.6% did not answer the question.

Participants reporting social needs were asked if they would like the research team to contact the clinic social work team on their behalf. 8.3% (18/218) requested this help and were referred to the social work team. These 18 participants represented 19.6% (18/92) of people reporting at least one need. Of the 92 participants reporting any need, nearly half (*n*=44) reported a single need, and these mainly involved housing.

Sociodemographic indicators show that the sample was broadly representative of clinic appointments in the study period, though less likely to include minorities (Appendix). In the social needs survey sample, 71% reported they were female, 78% reported white race-ethnicity, and the median age was 45. Among the full set of patients with appointments in the clinic during this period, 68.3% were female, 69.4% were white, and the median age was 45. In the social needs sample, 37.5% reported incomes less than $50,000 and 84% reported education beyond high school.

### Prevalence and correlates of social needs

Table 1 summarizes social needs reported by the patient sample. Of the five social needs, participants mentioned problems with housing (26.6%) and interpersonal threats (20.2%) most often. Food insecurity (15.6%) and difficulties with transportation (12.9%) followed. Problems with utilities were least common, with 5.5% reporting need.

**Table 1:** Social  need indicators

**Need**	**Comfort with question about need, % (n)**	**Reporting need, % (n)**	**Of patients reporting need:**			
			Asked in clinic, 12-mo % (n)	Discomfort when asked, % (n)	Want assistance, % (n)	Received assistance, % (n)
**Housing**	96.7 (203)	26.8 (58)	19.6 (11/56)	7.5 (4/53)	17.9 (10/56)	3.6 (2/56)
**Food**	97.6 (205)	15.7 (34)	20.6 (7/34)	3.1 (1/32)	47.1 (16/34)	11.8 (4/34)
**Transportation**	97.6 (205)	13.0 (28)	21.4 (6/28)	3.9 (1/26)	32.1 (9/28)	7.1 (2/28)
**Utilities**	98.1 (205)	5.6 (12)	16.7 (2/12)	0.0 (0/12)	41.7 (5/12)	16.7 (2/12)
**Interpersonal Safety**	96.7 (203)	20.4 (44)	35.7 (15/42)	5.1 (2/39)	4.9 (2/41)	0.0 (0/42)

*Unstable housing*: 1+ problem (pests, mold, lead, heat, oven, smoke detector, water leak) or unstable housing (anxious about losing home or homeless)

*Food insecurity*: Sometimes/often run out of food or food does not last until end of month

*Transportation need*: Lack of reliable transportation

*Intermittent utilities*: utilities shut off in past year

*Threat to safety*: Worried about safety, threatened with harm, or sometimes/often screamed at or cursed

Sample size ranges from *n*=209-216

The number of social needs ranged from 0 (57.8%) to 5 (1.8%) and differed by race and gender group. Non-white participants reported a greater mean number of needs (1.4 vs. 0.68, *p* < .01). People reporting non-conforming gender status (*n*=12) reported a greater number of needs (1.7) than males (0.5, *n*=56) and females (0.85), *p* < .01. Poorer health was associated with greater social need. The mean number (SD) of social needs among people reporting excellent, very good, or good health (*n*=181) was 0.6 (1.0) compared to 1.8 (1.5) among people reporting fair or poor health (*n*=37), *p* < .0001.

Patients on the whole welcomed social needs screening. As shown in Table 1, for each particular need, nearly everyone reported comfort with the inquiry. In a global question, 85% of patients reported they were very or somewhat comfortable with the questions. Even patients who reported a social need appreciated questions about their needs. Less than 10% of patients with a social need reported discomfort with questioning about the need. Discomfort with social needs screening was higher among African Americans (10.7%) than whites (3.2%) but that difference did not reach statistical significance (*p*=.07).

The number of social needs was related to socioeconomic status. Correlations were as high as -0.53 for income and -0.41 for the 10-point “best-off/worst-off” rating for SES ladder. Older age (*r*= -0.27) and greater education (*r*= -0.32) were associated with fewer social needs. Finally, perceived disrespect in the clinic (*r*= 0.38) and lower trust with health care providers (*r*= -0.30) were associated with greater needs. None of the SES indictors or measures of patient-physician relationship were significantly correlated with whether clinic staff asked about needs (*r*= -0.18-0.05).

Patients reporting a social need were asked additional questions about their comfort with questioning and support in addressing the need. Among patients reporting a social need in this encounter, 16.7% to 35.7% reported that clinic staff had asked about needs in contacts over the prior 12 months. Questions about interpersonal violence were most common. Patients overall were comfortable with such questioning; less than 7.6% reported discomfort with these questions. Requests for assistance with social needs were also common. Among people reporting needs, over 40% requested assistance to address food insecurity or access to utilities. Notably, despite its high prevalence (20.2%), only 4.9% reported need for assistance to address interpersonal violence, and none reported receiving help to address the need. Across the five needs, receiving assistance with utilities was most common, but even here only 16.7% reported any such help.

### Discomfort with social needs information included in the medical record

In this primary care patient sample, 20% (43/215) reported they were “somewhat” or “completely uncomfortable” having social needs information included in their medical record. 44.7% were “completely comfortable” with inclusion of social needs information. We examined the relationship between level of comfort and sociodemographic, health, patient-physician relationship, and social needs measures by estimating a multiple linear regression model (Table 2). In this adjusted model, number of social needs and level of trust in the patient-physician relationship were the only significant correlates. With each additional social need, discomfort increased by 0.19 (*p* < .05) on the 5-point rating. With each additional point of greater trust on the 10-point rating, discomfort declined by 0.16 (*p* < .01).

**Table 2:** Correlates of discomfort with social needs reported in EHR

**Correlate**	**Coefficient (SE)**	**95% Confidence Interval**
Age group	.09 (.05)	-.02, .19
Gender Identity		
Female, other	Ref	
Male	.15 (.16)	-.16, .47
Race		
Non-white	Ref	
White	.22 (.24)	-.24, .69
Education	.02 (.14)	-.25, .28
Self-rated health	-.08 (.10)	-.28, .13
Trust	-.16 (.06)**	-.28, -.04
Social needs (sum)	.19 (.09)*	.02, .37
Constant	3.02 (.92)***	1.21, 4.83

Model F, 2.52 (201 df), *p* = .017, Adj *R*^*2*^ = .05; *n*=202

Outcome: Discomfort (1=completely comfortable to 5=completely uncomfortable)

Correlates: age (7 categories, from 18-24 to 75+); education (high school, college, post-college); self-rated health (5 categories, from excellent to poor); trust (1-10); social needs (count, 0-5)

* *p* < .05, ** *p* < .01, *** *p* < .001

A simpler descriptive approach shows that discomfort with EHR documentation was not limited to people reporting a greater number of social needs. Among people reporting 0-1 social need, 15.4% (26/169) were uncomfortable or very uncomfortable with EHR documentation. Among people reporting 2-5 social needs, the proportion increased to 37.0% (17/46), *p* = .001.

## Discussion

CMS seeks to determine if addressing social needs has beneficial effects on health outcomes and health care costs [10, 11]. Social needs are more common among vulnerable populations (for example, patients with low income or low health literacy), as well as in patients with chronic diseases and mental health conditions [12]. Addressing social determinants as part of an expanded continuum of medical and human services should in principle improve patient health.

This study suggests that people are comfortable with questions about social needs in the primary care setting. Even patients reporting these needs express comfort with such questions. However, our results also suggest that people reporting a greater number of social needs are wary of having this information placed in the EHR. This effect persisted even when controlling for sociodemographic status, general health, and level of trust in patient-physician relationships. This is a concerning finding since one rationale for collecting social need data is to use this information, presumably tracked in the EHR, for addressing needs.

The prevalence of social needs in this sample was similar to reports from other adult primary care patients. For example, in a Kaiser study in which half the sample were covered through Medicare, 13.3% reported housing instability and 11.1% food insecurity. Over half of these patients wanted help to address the need [3]. Comparison of the prevalence of social needs across samples is challenging: “there is no consensus on how or how often patients should be screened for social needs, or which patients should be screened, in which settings, and by whom” [4]. Still, our finding that 20% of patients were very or somewhat uncomfortable with an EHR record of their social needs is in accord with prior research using a large sample across diverse clinical settings (19%) [9].

This research shows high levels of comfort with questions about social needs but less comfort with EHR documentation of such needs. Reasons for this difference are worth additional investigation. In this study we did not ask patients why they might be uncomfortable with such documentation. However, qualitative research from prior studies provides some insight. A subsample from one study [9] were asked about their perspectives on screening, including comfort with EHR documentation of social needs. Some patients were concerned about privacy and feared social needs information would be shared outside the health care team, while others doubted the utility of documenting such information [13]. Discomfort with EHR documentation of social needs reflects concern for privacy and fear of stigma but perhaps also low confidence that health care providers can actually use this information to help patients. Other patients may already receive help from other sources and may decline for that reason [5].

What would it take to make patients feel more comfortable with EHR documentation of social needs? Should we seek to increase this comfort level? Without adequate ways of helping patients find assistance, and without a way to use this information to deliver more effective care, the justification for collecting and documenting social needs in primary care is questionable. One potential intervention is to bring services designed to remediate social needs into the clinic, so that clinicians and social work teams can immediately connect patients to providers. The presence of services on site might destigmatize patient reporting of social needs and reassure patients that documenting these needs is an important step in addressing them. It may be possible as well to frame EHR documentation of social needs screening in positive ways, showing, for example, how a record of this information may be relevant for a patient’s care planning.

In addition, two trends suggest the value of direct elicitation of social needs information from patients. First, studies continue to show the value of eliciting social needs for outcomes important for patients, such as providing transportation to reduce missed medical appointments [14], providing housing to reduce COVID transmission [15], changing landlord behavior to reduce home health hazards [16], and using food prescriptions to address nutritional deficits [17]. Second, health care organizations and insurance plans are already acquiring social needs data by interrogating EHR records and geocoded public and proprietary databases. New efforts have also harnessed natural language processing of clinical notes in the EHR to identify social needs. In a Kaiser Permanante study, a comparison of housing and transportation needs identified by natural language processing showed moderate agreement with patient self-reported social needs [18]. Thus, combining direct elicitation of social needs and automated data mining efforts will likely give a fuller picture of the significance of social needs in health.

Limitations of this research include a relatively small sample, use of a single clinic, and possible effects of the COVID pandemic on medical care and trust in health care professionals more generally. The low response rate and potential selection bias limit generalizability. As Table 1 shows, missing data are also a potential concern as a small number of patients reporting needs did not complete additional questions on discomfort and assistance. Still, our findings suggest that patients with greater social needs were less comfortable with EHR documentation of these needs, and that in a subset of patients with no or only minor needs, some 15%, were also uncomfortable with this use of the EHR. Some of this discomfort may come from lack of familiarity with the EHR more generally. Patients may not be aware of the many other kinds of information routinely documented in the EHR, such as information about substance abuse, mental health diagnoses, number of sexual partners, sex of sexual partners, or hepatitis C status. Health care professionals ask about many potentially stigmatizing diagnoses and behaviors. It is not clear that “comfort” with EHR documentation of this information would be different from that of social needs.

In summary, this research shows patients in primary care are comfortable with questions about social needs but less comfortable with having this information placed in the EHR. Since documentation of social needs in the EHR will be important for the success of the proposed CMS quality measures related to social needs, it may be valuable to develop strategies to address this discomfort. These include framing EHR documentation of social needs screening in positive ways and linking primary care to services for addressing these needs.

## Data Availability

Deidentified data will be made available to qualified researchers upon submission of an analytic plan and request to the corresponding author (Steven M. Albert, smalbert@pitt.edu).

## Appendix

Sociodemographic Status of Patient Sample and Clinic Population

**Table:**

	**Patient Sample,** *n*=218	**Clinic Appointments in Study Period** *n*=18,692
***Gender-Sexual identity***		
Male	56	5,920
Female	150 (71%)	12,772 (68.3%)
Trans, nonconforming, other	5	
Missing	7	
***Age***		
18-24	14	
25-34	46	
35-44	36	
45-54	26	Median = 45
55-64	42	
65-74	20	
75+	22	
Missing	12	
***Race-Ethnicity***		
African American	32	3,508
White	165 (77.8%)	9,768 (69.4%)
Latino	5	100
Other	8	704
Missing	8	4,612
***Education***		
High school	33	
College	73	
Post-college	108	
Missing	4	
***Income ($)***		
0-5,000	7	
5,001-10,000	9	
10,001-15,000	3	
15,001-20,000	6	
20,001-25,000	6	
25,011-30,000	1	
30,001-35,000	8	
35,001-40,000	17	
40,001-50,000	12	
50,001-75,000	25	
75,001-100,000	23	
100,001-150,000	25	
150,000+	42	
Missing	34	

## Abbreviations

HER: Electronic Health Record
SDOH: Social Determinants of Health
CMS: Centers for Medicare and Medicaid Services
AHC: Accountable Health Communities
HRSN: Health-Related Social Needs
SIREN: Social Interventions Research & Evaluation Network
AHC: Accountable Health Communities
